# Acid stress response in *Saccharomyces cerevisiae *involves ionic homeostasis and calcium signaling pathway

**DOI:** 10.1186/1753-6561-8-S4-P193

**Published:** 2014-10-01

**Authors:** Marcos Vinicius Almeida, Júlio César Rosa, Heloá Queirós, Rogelio Brandão, Ieso Castro

**Affiliations:** 1 Laboratório de Biologia Celular e Molecular, NUPEB, Universidade Federal de Ouro Preto, Ouro Preto, Minas Gerais, Brasil

## 

To maintain their productive capacity, yeasts cells need to adapt to environmental changes that occur during production process. Acid stress response is of interest, because such exposure occurs under industrial conditions, such as the acidic treatment before cell recycling in fermentation process. Resembling the response to weak organic acids, resistance to inorganic acids exposure involves changes in the membrane conductivity to H^+^, active extrusion of acid from the cell and gene expression modulation. We previously investigated cellular tolerance to HCl pH 2,0 and suggested that the systems involved in the maintaining of the plasma membrane potential (PMA1p H^+^-ATPase and secondary transporter systems) were linked to the acid stress response [1]. The present study focused on plasma membrane H^+^-ATPase participation and calcium signaling events observed in response to acid stress. To evaluate cell viability, yeast strains were grown in YPD medium up to OD_600nm _1,0. Cells were harvested by centrifugation and washed with YP medium. They were suspended in HCl aqueous solution (pH 2.0) supplemented with 86mM NaCl and incubated in orbital shaker at 30°C. Aliquots were collected at 0, 10, 30 and 60 minutes, washed, diluted and spotted onto YPD-agar. Colonies were counted after 48 hours. To determine cell buffer capacity, cells were grown and harvested as described above and exposed to acid pulses with small volumes of HCl. Resultant pH were measured with a pH meter. *ENA1 *gene was cloned in a single copy plasmid, p417CYC, and in a multicopy plasmid, p427TEF. Strain LBCM479 (*ena1-4Δ*) was transformed with both constructions and H^+^-ATPase activity was measured as previously described [2], before and after a pulse acid. Cytosolic calcium flow was monitored by the aequorin method [3] with some modifications. Yeasts carrying the apoaequorin-expressing plasmid, pVTU-AEQ, were harvested, washed with MES/Tris 0,1M buffer and incubated with 50 μM of coelenterazin to reconstitute the functional aequorin. Cells were transferred to a luminometer tube. Light emission was monitored for 1 minute before and lasting until 10 min after acid pulses. Our results show that the extracellular buffering capacities of *S. cerevisiae *W303 and *S. boulardii *(probiotic strain) were similar, but *S. cerevisiae *W303 was more affected by acid pulses than *S. boulardii*. This yeast had a higher total buffer capacity and greater ability to maintain pH homeostasis after acid stress than *S. cerevisiae *W303. Activation in vivo of the PM H^+^-ATPase by a pulse acid showed a correlation between ENA1 levels and the acid-induced PM H^+^-ATPase activation. Acid pulses triggered very low calcium signals compared to glucose pulses. KCl pulse was used as negative control. The acid-induced transient elevation of cytosolic calcium was pH dependent and confirmed by the fluorescent calcium indicator Oregon Green^® ^488 BAPTA-1, AM. Additionally, viability assays show that calcineurin and Crz1p are required to induce the acid-stress response. In conclusion, the present study shows that the internal pH of yeast is regulated by several systems, including the plasma membrane H^+^-ATPase, and that Ena1p has an important but undefined role in the cellular response to acid. We also demonstrated that the acid stress response is dependent on calcium signaling pathway.

## References

[B1] Sant'Ana GS Paes LS Paiva AFV Fietto LG Tropia MJM Giunchetti DSL Lucas C Fietto JLR Brandao RL Castro IM Protective effect of ions against cell death induced by acid stress in Saccharomyces FEMS Yeast Res 2009 9 701 712 10.1111/j.1567-1364.2009.00523.x 19473262

[B2] Becher dos Passos J Vanhalewyn M Brandao RL Nicoli JR Thevelein JM Glucose-induced activation of plasma membrane H+ -ATPase in mutants of the yeast Saccharomyces cerevisiae affected in cAMP metabolism, cAMP-dependent protein phosphorylation and the initiation of glycolysis BiochimBiophys Acta 1992 1136 57 67 10.1016/0167-4889(92)90085-p 1322708

[B3] Tisi R Baldassa S Belotti F Martegani E Phospholipase C is required for glucose-induced calcium influx in budding yeast FEBS Lett 2002 520 133 138 10.1016/S0014-5793(02)02806-5 12044885

